# Fetal alcohol spectrum disorder and Risk-Need-Responsivity Model: A guide for criminal justice and forensic mental health professionals

**DOI:** 10.3389/fpsyg.2022.689837

**Published:** 2023-01-27

**Authors:** Jerrod Brown, Joe Arvidson, Megan N. Carter, Vanessa Spiller

**Affiliations:** ^1^American Institute for the Advancement of Forensic Studies, St. Paul, MN, United States; ^2^Concordia University, St. Paul, MN, United States; ^3^Metropolitan State University, St. Paul, MN, United States; ^4^American Institute for the Advancement of Forensic Studies (AIAFS), St. Paul, MN, United States; ^5^University of Washington, Seattle, WA, United States; ^6^Department of Social and Health Services, Special Commitment Center, Steilacoom, WA, United States; ^7^JumpStart Psychology, Brisbane, QLD, Australia

**Keywords:** fetal alcohol spectrum disorder, risk need responsivity model, criminal justice, forensic mental health, recidivism

## Abstract

Fetal alcohol spectrum disorder (FASD) is an umbrella term used to describe a range of significant neurodevelopmental, brain-based disorders and impairments that result from prenatal alcohol exposure. FASD is a high prevalence but underdiagnosed group of disorders affecting between 17 and 36% of individuals in criminal justice settings. Despite being a high-impact disorder associated with lifelong impairments with a significant need for services and interventions, little research has been completed on how to best support individuals with these conditions in criminal justice settings. This article proposes a renewed focus on applying and adapting the Risk-Need-Responsivity (RNR) approach to individuals with FASD in criminal justice settings. This will assist in better determining the needs and interventions likely to effect change and reduce recidivism for this prominent criminal justice-based population. The RNR approach has been used with multiple corrections populations to determine the need and most appropriate interventions, as well as how to best allocate scarce resources. As the prevalence of FASD becomes better understood and recognized, evidence-based approaches to addressing this specific sub-population are necessary to effect change and reduce recidivism and ongoing involvement in the criminal justice system.

## Fetal alcohol spectrum disorder

Fetal alcohol spectrum disorder (FASD) describes the pervasive cluster of severe neurodevelopment impairments which can arise from prenatal maternal alcohol exposure (Streissguth et al., 1996; May et al., 2018; Pervin et al., 2022). Individuals with this condition can experience impairments across a range of different brain-based domains including cognition, memory, executive functioning, affect, and adaptive functioning (Cook et al., 2016; Mattson et al., 2019). To add to the complexity of this life-long and complex disorder, the brain domains impacted can vary from individual to individual, making it near impossible to identify a specific profile that typically characterizes the condition.

The authors of this article have a collective experience of several decades of working and living with those who have or are suspected of having FASD. This collective experience includes clinical treatment settings, forensic assessment and treatment settings, caregiving of those with FASD in a professional role and a parental role, as well as research specific to the various consequences of prenatal alcohol exposure. Additionally, some of the authors have expertise in the Risk, Needs, and Responsivity model and the use of this model with complex populations. Through this vast collective experience, the authors are providing a proposal of the use of the Risk-Need-Responsivity Model as it would be understood in relation to FASD, as well as practical application information and implications for the RNR model with those who have FASD to reduce recidivism and involvement in the criminal justice system. It builds upon previous work by authors such as Pei and Burke (2018), by providing practical examples of each of the elements in the RNR model and how this may manifest for individuals with FASD. This includes a literature review of both the RNR model and FASD to understand how the two areas of focus can be understood. Additionally, this article is provided to promote further research on the RNR model for those diagnosed with or suspected of having FASD and to encourage training on and implementation of a model that may properly support the complex needs of this under-recognized and over-represented population in the criminal justice system.

The prevalence of FASD in the general population varies between 1 and 10% in Western countries (Roozen et al., 2016; Lange et al., 2017; Shölin et al., 2021), but this prevalence increases dramatically to between 17 and 36% in correctional settings (Fast et al., 1999; Bower et al., 2018; McLachlan et al., 2018, 2020; Popova et al., 2021). Although specific facial features are commonly recognized as being associated with prenatal alcohol exposure, the majority of individuals exposed to alcohol prenatally do not exhibit these associated facial features, further contributing to the underdiagnosis and lack of identification of FASD (Popova et al., 2021). With severe impairments in executive functioning including difficulties linking cause and effect, poor organizational skills, and high levels of impulsivity accompanied by deficits in other areas such as problem solving, social skills, attention, and memory, it is not difficult to see how individuals with FASD might find themselves disproportionately represented in criminal justice populations. Such impairments are likely to contribute to individuals engaging in behaviors that lead to incarcerations but then impact significantly on their participation and understanding of legal processes once in the system. If not addressed, the neurodevelopmental impairments that contributed to their incarceration remain unchanged throughout their time in custody causing further difficulties and increasing their chances of recidivism upon release. The likelihood of this occurring is exacerbated when proper services are not in place (Wartnik and Brown, 2016). In fact, these same deficits make individuals with FASD vulnerable to other risks while involved in the criminal justice system (e.g., learning maladaptive coping skills, misunderstanding expectations, and an inability to remember rules resulting in infractions/violations, poor interpersonal boundaries, and victimization).

When interviewing individuals in the criminal justice system, knowledge of potential indicators, or “red flags,” of FASD is helpful to determine if more specific screening should be completed. In addition to the above-described deficits (e.g., executive function deficits, communication problems, impulsivity, etc.), there is historical information that is important to note. A few potential indicators of FASD include (but are not limited to): maternal history of significant alcohol and/or substance use; history of out-of-home placement as a child; history of ADHD or learning disorder/ special education; specific deficits in math; history of seizures; history of growth problems; lower intellectual functioning; hyperactivity; stubbornness; irritability; risk-taking; problems following multiple-step directions; problems attending appointments; inability to manage money; inappropriate affect; and appears socially and developmentally younger than their chronological age (except for some types of speech in which they can appear superficially proficient).

Although the question of competency to stand trial for persons with FASD has been raised (Brown et al., 2017b, 2018), the suitability to undergo assessment and to have one’s case plan formulated without adequate FASD considerations has not (Douds et al., 2013). In contemporary corrections in the United States, the level of services provided to individuals during incarceration as well as during probation or parole in the community may be determined through use of the Risk-Need-Responsivity Model (Wormith and Zidenberg, 2018). Understanding this model and its application to individuals with FASD is key to ensuring that appropriate service provision occurs, ultimately increasing the chances of “successful” sentence completion and reducing recidivism.

## The Risk-Need-Responsivity Model

The Risk-Need-Responsivity (RNR) model was developed by Andrews et al. in the 1990’s in order to provide an evidence-based framework for the assessment and interventions offered to offenders. Overall, this model aims to tailor the intensity of interventions for detainees based on an accurate assessment of the risk factors contributing their offending behaviors and their areas of need and then addressing these, thus reducing recidivism. Their approach to the assessment and treatment of criminal offenders is widely considered the premier model of supervision for correctional agencies around the world (Andrews et al., 2011; Looman and Abracen, 2013; Gearhart and Tucker, 2020). The model is comprised of three core principles: The Risk Principle, the Criminogenic Need Principle, and the Responsivity Principle.

The *Risk Principle* asserts that treatment and intervention resources should be allocated in accordance with the likelihood that a given person will recidivate, with more resources being allocated to “high risk” individuals as determined by the use of a validated risk assessment tool, and fewer resources being allocated to “low risk” individuals. This was a shift from inflexible blanket approaches previously adopted by probation and parole officers. Prior to this, the frequency of client contacts and the degree of resources directed to an individual client were based on the offenses they had committed (e.g., sexual offense, burglary, and assault) rather than an assessment of their recidivism risk and individual needs. The allocation of resources in this way has since been found to be problematic as underlying criminal behavior does not factor strongly into the prediction of risk. Andrews and Bonta (2006) found that allocating resources to “low risk” individuals with the aim of reducing recidivism while being not only ineffective may, in fact, increase recidivism risk as a result of a range of factors such as increased exposure to high-risk offenders. For example, under previous supervision approaches, a low-risk offender who is employed does not have drug and alcohol dependence issues, and has strong family and community supports may have been required to attend a twice-weekly cognitive behavioral interventions (CBI) group as a result of their offending behavior. That attendance would take them from paid employment and disrupt their existing supports, placing them in an environment with much higher-risk peers. Aside from learning the course curriculum, participants may also be influenced by the antisocial attitudes and behaviors of other group members. Therefore, when the Risk Principle is not implemented, agencies may be expending finances to inadvertently increase recidivism due to allocating and supervising low-risk, medium-risk, and high-risk individuals to ineffective levels and types of intervention.

When applied appropriately, the Risk Principle is more akin to approaches common in other fields such as a medical triage. Hospital emergency rooms are very adept at triaging patients and allocating those with the highest level of medical need to immediate and intense intervention while deprioritizing those with less severe, non-life-threatening conditions. If the same principles are applied to the corrections setting, clients with the highest risk of re-offending would be given priority in regard to supervision and resources such as programing. Those with lower risk of recidivism would still receive services but at a lower intensity.

The second factor to consider in the Risk-Need-Responsivity Model is that of *Criminogenic Need Principle*. The concept of need postulates that each individual has their own unique range of risk factors that contributed to their offending. If these risk factors are identified and managed, then offending rates and recidivism will decrease. Risk factors will vary from individual to individual and it is these risk factors that should be targeted by interventions. Low-risk offenders will have fewer criminogenic needs and, therefore, will require fewer resources. However, accurate assessment is needed in order to determine this and to appropriately triage the level and type of intervention most appropriate.

Criminogenic needs can be thought of as ‘drivers of crime.’ Accordingly, the Need Principle asserts that the most effective way to promote reductions in recidivism is to concentrate efforts into those variables which correlate most strongly with criminality, steering clear of non-criminogenic variables. Through meta-analysis of a wide range of correctional treatment programs, variables correlating most strongly to re-offending have been identified and clustered into eight different domains or silos, often referred to as the *Central Eight*. These are reflected below in Table 1 (Bonta and Andrews, 2007).

**Table 1:** The Central Eight Risk/Need Factors.

Criminogenic variable	Synopsis	
Criminal History	One of the best predictors of future behavior is past behavior. This entails a variety of criminal activity in multiple settings. Included within this domain would be the volume of offenses as well as documented rule infractions while on correctional supervision (e.g., probation violations). Although one’s history cannot be altered, a high-risk score in this area is reflective of low self-regulation.	
Pro-criminal attitudes	Within this domain are variables such as an individual’s core values and beliefs. Risk is typically reflective of attitudes favorable to crime and anti-social behavior. This can manifest itself *via* rationalizations, minimizations, and making favorable comparisons. Examples include, “They have insurance,” “nobody got hurt,” “everybody in this neighborhood carries a gun.”	
Pro-criminal associates	This domain does not merely focus on the presence of anti-social friends and associates, but also a void when it comes to pro-social connections.	
Antisocial Personality Pattern	This domain captures characteristics such as adventurous pleasure seeking, impulsivity, a callous disregard for others, and being restlessly aggressive.	
Family/Marital	Core in assessing risk in this domain is an examination of the quality of the relationship in question. What are the rules and expectation which have been established as to delinquent behavior? What is the strength of the bond in question?	
School/Work	This domain considers the quality of the satisfaction from school/ work, their ability to succeed in these settings, as well as interpersonal relationships within the setting of either school or work. Higher risk is determined when there are lower levels of performance and satisfaction with school or work.	
Substance Abuse	Consider not just past but current issues with alcohol and/or drugs. Current issues tend to be more predictive than past issues, although both carry weight in this domain.	
Leisure/Recreation	This category focuses on a lack of involvement in prosocial leisure pursuits. These tend to buffer against risk and augment positive connections.	

According to this theory, the greater the number of criminogenic needs targeted successfully, the greater the impact on decreasing subsequent recidivism. This also speaks to the necessity of accurate assessment. Well-intentioned and poorly informed practitioners may design inadequate or misdirected treatment/ intervention plans due to inaccurate assessment. For example, if an individual is at risk to re-offend due to the fact that they are high in the domains of alcohol/drug use and negative companions, addressing those areas of need will result in a correlating decrease in the risk of re-offending. However, if non-criminogenic needs are targeted, the risk of recidivism increases. Unfortunately, popular targets of change, such as trauma and low-self-esteem, are non-criminogenic. The probability of recidivism also increases by way inaccurate assessment. For example, assigning risk where there is none, or not assigning risk when it is warranted.

In addition to an assessment of the individual’s risks, identifying protective factors can also assist in a more thorough and accurate assessment. Protective factors, such as a positive and supportive family, stable employment, stable housing, community involvement, etc. may provide a base for managing risk factors and a strengths-based approach to treatment and supervision planning. Protective factors can then be developed or enhanced in addition to targeting risk factors for reduced recidivism.

The *Responsivity Principle* is arguably the most elusive of the three principles in part because it is actually comprised of two subcomponents. General responsivity indicates that cognitive-social learning techniques are the most effective in dealing with criminogenic needs. Specific responsivity speaks of tailoring the delivery of services to match the individual characteristics of the client. Specific responsivity can be thought of as “creating the ideal learning environment” for each individual (Bourgon and Bonta, 2014). This is particularly important in considering the specific deficits (as described above) for individuals with FASD, including learning problems, communication challenges, information processing deficits, social skills deficits, and executive functioning deficits.

To date, the Responsivity Principle remains the least researched of the three core principles of the RNR model (Cohen and Whetzel, 2014). This is particularly true with specialty populations, such as those with FASD, that have little direct evidence-based intervention research (Pei and Burke, 2018). Although the Responsivity Principle has traditionally been viewed as more of an afterthought by practitioners, non-adherence to this principle results in practitioners ignoring sound cognitive behavioral interventions and/or delivering services in such a way as to not resonate with the client’s learning style, cultural needs or necessary accommodations (Bonta et al., 2011).

The more that the RNR principles are adhered to in practice, the further recidivism risk decreases (Bonta and Andrews, 2007). This model has not only been found to be useful in having an impact on recidivism amongst adult male offenders, but also in preventing community violence (Dowden and Andrews, 2000), sexual offending (Hanson et al., 2009), institutional infractions (French and Gendreau, 2006), gang involvement (DiPlacido et al., 2006), young offenders (Andrews et al., 1990; Dowden and Andrews, 1999a), women offenders (Dowden and Andrews, 1999b), and mentally ill offenders (Andrews et al., 2001). When implemented correctly, adherence to the RNR model has been found to result in a decrease of up to 35% in recidivism rates (Bonta and Andrews, 2007). Conversely, the research reflects that non-adherence to the principles of Risk, Need, and Responsivity goes beyond ineffectiveness. Indeed, it can be harmful to client outcomes (Lowenkamp and Latessa, 2002).

## Applying the RNR model in FASD

Little research has been completed with regard to appropriate interventions in general for those with FASD (Pei and Burke, 2018; Shölin et al., 2021). Given the unique symptomatology and sequelae of FASD (e.g., often with an IQ higher than those with intellectual disability but with adaptive functioning deficits in the extremely low range making standard screening less likely to detect their deficits) and the disproportionate number of individuals with FASD or suspected of having FASD involved in the criminal justice system, examination of the application of the RNR model specifically to people with FASD is warranted and yet has been largely neglected in research. In theory, applying the RNR model to inherently high-risk populations may significantly decrease recidivism by improving assessment and treatment efficacy. However, the current research literature on this diagnostic group is limited with little information available regarding accurately assessing risk, identifying criminogenic needs, and in relation to the overall application of the RNR model to individuals with FASD (Pei and Burke, 2018).

With regard to the Risk Principle, longitudinal research is needed to establish risk and protective factors for different forms of recidivism in persons diagnosed with FASD. The absence of a risk factor, if replaced by its pro-social doppelganger, can be considered a protective factor. That is, if in the absence of negative peers, there is a determination of prosocial friends and associated, that area of one’s life can be considered a protective factor. If rather than a checkered history around employment, one has a healthy job history that can be deemed a protective factor. These protective factors can act as a buffer against risk, and should be augmented *via* the case planning process. Rather than simply endeavoring to extinguish risk, the practitioners should also build protective factors in their client’s lives. To be clear, the absence of a single risk item does not automatically equate to a protective factor. Rather, if the practitioner feels that in considering an entire criminogenic domain, i.e., family, that there exist exceptionally positive circumstances, then these factors, by virtue of their presence, may serve as protective factors. That is, if in the absence of negative peers, there is a determination that the attachment to the prosocial peers is sufficiently strong enough to serve as a model for prosocial behavior, it may be considered a buffer against risk. Research suggests that individuals diagnosed with FASD are more likely to demonstrate the *Central Eight* risk factors, including: A history of criminal behavior, antisocial cognitions, antisocial peers, antisocial personality pattern, family and/or marital discord, poor school or work performance, few positive leisure or recreational activities, and substance abuse (Conry et al., 1997; Streissguth, 1997; Bonta and Andrews, 2007; Rasmussen and Wyper, 2007; Spohr et al., 2007; Fast and Conroy, 2009; Rogers et al., 2013). Research may reveal additional risk factors linked specifically to the underlying deficits associated with FASD. Pei and Burke (2018), for instance, describe difficulties with executive functioning, including inhibition, decision-making, working memory, integration of information, and cognitive flexibility as possible sources of risk. However, the risk profile of individuals with FASD will vary according to the unique patterns of brain injury associated with prenatal alcohol exposure found in each individual with FASD. Thus, an individualized and thorough assessment of a person’s risk is key to appropriate planning using the RNR model. Novick Brown and associates (Fabian, 2021) have provided specific information about assessing individuals with FASD in forensic settings, including the use of risk assessment measures. Specifically, Fabian (2021) reports that some risk assessment measures allow for clinicians to account for prenatal alcohol exposure under categories such as “major mental disorder” while other measures have no such consideration. Fabian further comments that certain risk measures that do not account for neurodevelopmental disorders, intellectual disability, and neuropsychological impairments in executive functioning may be inappropriate for use with those with FASD, as those measures “are not empirically equipped to assess violence risk…” in such individuals (p. 359). Common risk assessment measures may fail to capture variance that is specifically related to FASD, which may result in underestimation of risk and misalignment in treatment and management plans that fail to account for specific needs that could be otherwise addressed (Pei and Burke, 2018). Further research specific to this issue is necessary.

Information on *protective factors*, which directly reduce or moderate the likelihood of future recidivism (e.g., self-esteem, high intelligence, strong social support, and problem-solving abilities), should also be measured (Turner et al., 2007). While not necessarily validated specifically for an FASD population, some possible measures of protective factors may include the Structured Assessment of Violence Risk in Youth (SAVRY; Borum et al., 2006) or the Structured Assessment of Protective Factors (SAPROF; De Vogel et al., 2009), available in both adult and youth versions. Both measures have been studied with many different populations and may assist with structuring an assessment of individuals with FASD, although additional research needs to be conducted to determine the applicability of these measures with this population and if there are additional relevant factors specific to FASD not included in these measures.

Multivariate modeling studies may be able to identify a parsimonious set of factors which best predict recidivism risk in individuals with FASD. This set of factors can then be cross-validated on new samples and the model’s predictivity, validity, and reliability compared to available “gold standard” recidivism risk/needs assessment tools, including the Level of Service/Case Management Inventory (LS/CMI; Andrews et al., 2004), the Federal Post-Conviction Risk Assessment (PCRA; Johnson et al., 2011), and the Ohio Risk Assessment System (ORAS; Latessa et al., 2009). For a review of the most commonly used and best-validated recidivism risk/needs assessment tools, see the handbook by Hamilton et al. (2018). To date, none of these instruments has been validated on adult offenders diagnosed with FASD, although promising preliminary findings have been discovered for the use of risk/needs assessment tools with young offenders with FASD (McLachlan et al., 2018). Using scientific processes to identify accurate and reliable risk and protective assessment procedures is a critical step in effectively applying the RNR model to persons diagnosed with FASD.

With regard to the Need Principle, further research is needed to establish best practice investigative interviewing guidelines for individuals diagnosed with FASD. As our team has discussed elsewhere (Brown et al., 2016; Watts and Brown, 2016), this clinical population is vulnerable to suggestibility and confabulation such that false testimony and even false confessions can be unintentionally provided to interviewers (Brown et al., 2022). These issues are important to consider when assessing an individual’s needs, to ensure accurate assessment and planning for responsivity. *Suggestibility* is a personality trait with a cognitive component which makes an individual susceptible to being manipulated in various contexts, whereas *confabulation* refers to the unintentional fabrication or distortion of memories (Fotopoulou et al., 2007; Brown, 2017). For clinicians, this means using FASD-informed interviewing techniques, collecting information from collateral contacts, and optimizing the interviewing environment to minimize pressure is critical to obtaining truthful responses which can then be used to make diagnostic and treatment decisions.

In the authors’ experience, although methods of interviewing during a risk assessment constitute a portion of forensic training, FASD and the phenomenon of confabulation and suggestibility are rarely emphasized, if covered at all. Often times, the criminal justice practitioners view themselves more as a collector of information than a client-centered assessor endeavoring to formulate the ideal case plan for their client. It is often noted that the more the assessment interview resembles an interrogation, the less accurate it will be. This may increase the risk of suggestibility. According to Gudjonsson (1987) interrogative suggestibility can take place when the interviewee is being interviewed about recollections and experiences from their past by a person in a position of authority, and when the interview occurs within the context of a closed and stressful social interaction (Gudjonsson and Clark, 1986; Gudjonsson, 1987).

Unfortunately, limited research has been conducted on suggestibility and confabulation in persons diagnosed with FASD in the criminal justice system, despite the fact that obtaining incorrect information about a client’s offense chain (i.e., the series of events which led up to the index offense being committed) means that the person’s risk may be incorrectly assessed and the Need Principle will not be adhered to. In addition, further research is necessary to determine if and how clinical and forensic-based interviewing approaches need to be modified to be of greatest utility when interviewing individuals diagnosed with FASD.

With regard to the Responsivity Principle, research has not been conducted to evaluate which symptoms of FASD addressed during the assessment and treatment process most improves outcomes. Although this principle is the least commonly addressed of the RNR model (as noted above), it is arguably the most important for this diagnostic population, given the numerous cognitive and adaptive impairments associated with prenatal alcohol exposure. Given the over-representation of this population in the criminal justice system, addressing the Responsivity Principle adequately for those with FASD is essential to supporting this population in reducing further criminal justice contacts and alleviating the burdens on a system that is currently ill-prepared to effect change in individuals with FASD.

A first step in better understanding this principle with individuals with FASD would be to first investigate the factors which may limit or enhance responsivity in individuals with FASD. Recall that General Responsivity calls for the use of cognitive behavioral approaches to deal with criminogenic need. Whereas Specific Responsivity can be thought of as tailoring the delivering of interventions in a manner which creates an optimal learning environment (Bourgon and Bonta, 2014). For example, traditional, large, talk-based groups may not be as effective with individuals who have FASD who process information more slowly, struggle with effective communication, and have lagging social skills. It would be erroneous to assume that the factors which enhance and decrease responsibility in the general population are the same factors that apply to those with atypical brain development and brain-based injuries. Secondly, based on a greater understanding of the aforementioned factors, the application of cognitive behavioral approaches to individuals with these issues is needed but traditional approaches may not be as effective with this population (Verbrugge, 2003; Burd et al., 2010). Certain patterns of impairment, for example those with below average IQ’s or those with high levels of emotional dysregulation and impulsivity, may not respond to cognitive behavioral approaches in the same way as individuals without those impairments despite still meeting criteria for the same diagnosis. Unfortunately, FASD has a highly individualized pattern of impairment, making broad generalization difficult and access to more individualized services essential.

As previously noted, the general Responsivity Principle indicates cognitive-social learning techniques are the most effective at addressing criminogenic needs. In practice, these approaches have been the most frequently studied but rarely have they been examined on young people with severe neurodevelopmental impairment. Given that cognitive and adaptive impairments are required to obtain a FASD diagnosis, further exploration of this at the broader diagnostic but then individual levels are required. Despite this, with or without diagnosis, individuals with FASD will likely be directed into cognitive behavioral interventions due to a lack of viable alternatives and limited system resources.

If these sequelae of severe neurodevelopmental impairment are not taken into account, the suggestibility and confabulation characteristic of this population may result in inaccurate assessments and intervention recommendations due to the over- or under-endorsement of both historical as well as clinical factors (e.g., the identification of psychopathic traits such as pathological lying, not taking responsibility for actions, or impulsivity when these are often behavioral symptoms of underlying brain injury characteristic of FASD rather than characterological traits). It is important that clinicians working with this population for both assessment and intervention, have specialized knowledge and training to best serve the needs of those with FASD. Indeed, the entirety of the RNR model arguably rests on accurate assessment of criminogenic need and responsivity issues. Although not originally incorporated into the RNR model, assessment of protective factors has more frequently become expected as a part of comprehensive assessment. However, more research is needed on the impact of protective factors in the RNR model generally and more specifically with regard to the application to those with FASD.

Informed interviewing involving individuals with an FASD is very important. Risk assessment interviews are not merely questionnaires completed by corrections personnel. It is an opportunity to gather additional important information about the individual such as the person’s ability to process information, understand and communicate language, demonstrate memory abilities, attend to and answer the question asked, demonstrate their ability to provide a coherent narrative, etc. There is a recognized risk of false confessions in individuals with FASD, increased even more when certain interview styles are implemented. Their impairments (e.g., communication deficits, inability to anticipate consequences, tendency to be overly trusting with authority, eager to please when in a stressful situation) may result in improperly obtained confessions on the part of the police or other criminal justice professionals (Allely and Mukherjee, 2019). Corrections officials, probation and parole officers, forensic clinicians, and others conducting risk/need assessment with a FASD client may be in a sense improperly obtaining false confessions while attempting to ascertain their client’s criminogenic needs. Within the realm of criminal justice, persons with FASD are readily manipulated by others (Greenspan and Driscoll, 2016). Although the degree of overt manipulation on the part of the assessor during the assessment interview process is unknown, any degree of unconscious bias could be aggravated with an FASD client, who is more susceptible to manipulation.

To decrease bias and unintentional manipulation, more research is needed to determine the degree of FASD awareness in probation and parole officers, those most likely to conduct risk/need assessments for their caseloads. One indication that there is a dearth of knowledge in the criminal justice field in this area is a survey of public defenders, which revealed that less than 20 % of respondents reported having received training as to the impairments experienced by their FASD clients (Brown et al., 2017a). Other studies of the knowledge about FASD in criminal justice professional populations also indicate a significant lack of training in this area (Brown and Singh, 2016; Mutch et al., 2016; Brown et al., 2019). This lack of FASD knowledge significantly impacts the criminal justice professional’s ability to identify the necessary information for adequately implementing the RNR model with this population.

## Fetal alcohol spectrum disorder as assessed in the context of RNR

From the perspective of the FASD population, it may be beneficial to look at the symptoms commonly associated with FASD (e.g., executive functioning deficits, adaptive functioning deficits, academic/learning deficits, and self-regulation deficits) as a starting point, as they relate to the RNR model. By examining the various brain domain impairments of FASD, a more in-depth analysis may be fostered. One such brain impartment is that of executive functioning. Deficits associated with executive functioning may be reflected in the criminogenic needs of Criminal History, Pro-Criminal Attitudes, and Anti-Social Personality Pattern. Low self-regulation and impulsivity are captured within the domain of Criminal History in RNR risk/need assessments, yet an individual’s criminal history is traditionally viewed as a “static” factor.

Another impairment of FASD is that of Adaptive Functioning. This may result in difficulties with reading and writing, which of course would be captured in the criminogenic domain of Education/Employment. Cognition, another FASD brain impairment, could be linked once again to the criminogenic domains of Pro-Criminal Attitudes and Anti-Social Personality Pattern. Cognitive deficits could also be aligned with deficits around Education/Employment.

The following provides a summary of the recognized areas of impairment that are common in individuals with FASD (Bower and Elliott, 2016) and how these may impact on accurate assessment as well as suitability for interventions. Each of these areas of impairment, typically manifested to some degree by those with prenatal alcohol exposure, should be assessed as part of the Risk Principle and then accommodated within the planning for the Needs and Responsivity Principles. Because each individual will manifest these impairments differently, as noted above, individualized planning using the RNR model will be most effective to address these impairments.

## Implementation and training

Although establishing a correctional assessment and treatment workflow following the RNR principles is an important first step in establishing appropriate interventions to reduce recidivism among individuals diagnosed with FASD, the simple implementation of such a workflow does not mean that it will be adhered to in practice. This lack of implementation *fidelity* is commonplace for a variety of reasons including lack of program funding, lack of trained staff, poor cooperation from the individual, etc. This can result in risk/needs assessment tools being administered but not used to target criminogenic needs in a responsive manner, rendering the tools under- or ineffective. For example, Singh et al. (2013) found that a minority of risk factors identified using a risk/needs assessment tool were actually addressed in treatment for adolescent offenders in the United States. For a literature review of studies on the assessment of fidelity and methods of maximizing it, see the work of Mowbrey et al. (2003). Furthermore, the application of assessments in these settings is particularly problematic as it gives the perception that these factors are being taken into account in treatment planning when, in fact, they are not.

In order to faithfully implement the RNR principles into correctional practices, evidence-based training programs are needed (Dyck et al., 2018). Research has found that such training programs can enhance clinicians’ perceived confidence in conducting risk/needs assessments, knowledge about available assessment tools, and documentation of recommended interventions, especially if training is multimodal (McNiel et al., 2008; Reynolds and Miles, 2009; Storey et al., 2011). Supervised practice assessing for and developing appropriate interventions may assist the real-life application of the RNR model. Reynolds and Miles (2009) have also identified that training delivered by both qualified as well as trainee staff is just as effective in increasing the quality of risk/needs assessments.

Any such training should be supplemented by in-person or online continuing education in the signs and symptoms of FASD, and how they may impact the effectiveness of otherwise evidence-based assessment and treatment techniques. In addition to stressing the importance of assessors being cognizant of FASD in the populations they serve, staff need to be made aware of the various recommendations to best factor this information into their practices. Studies have found that providing “booster” sessions every 2–3 years following an initial training can be helpful in knowledge retention (Hamilton et al., 2018). These boosters may involve practice examples such as showing participants a recorded mock interview, allowing them to evaluate the interviewee, and then reviewing their evaluations. There are strategies which can be utilized in the interview process for the criminal justice professional to embrace to improve information gathering (Brown et al., 2016), and these same practices could be extended to the risk assessment interview process. The probation officer conducting a risk/need assessment on an individual with suspected or confirmed FASD should be aware of the fact that their client will generally function at a higher level if interviewed in a structured and predictable environment. This includes minimizing stimuli and distractions as well as tailoring the questions to the person’s developmental abilities.

## Best practice assessment interviewing

When conducting a risk assessment interview with an individual with diagnosed or

suspected FASD, it is considered a best practice in risk assessment interviewing to begin the

process with a structuring statement. This statement should include the following:

1. Note the purpose of the interview, explain that collateral information will be collected, and advise that if interested, the client can receive the assessor’s feedback.
2. Describe how the client will be asked a series of information-gathering questions.
3. Explain how the interview will conclude, such as follow-up questions or forms filling in any gaps.

This structure is particularly important for individuals with FASD who often lack the.

understanding of the purpose of why an interview would be conducted in this setting and who cannot intuit this structure themselves. Without a clearly articulated structure, the FASD client may easily become confused and overwhelmed which may present as anger, withdrawal, frustration, or irritability, and behavioral symptoms which could be misinterpreted as risk factors. Sufficient time must be given to persons with FASD to process what is being asked of them so that they may have time to generate a coherent and organized response (Brown et al., 2020).

Table 2 provides a range of strategies that assist in conducting a risk assessment for an individual with diagnosed or suspected FASD (Brown et al., 2014, 2016), specifically in order to reduce the risk of confabulation and suggestibility. Individuals with FASD have increased vulnerability to both confabulation and suggestibility, as previously mentioned, and reducing the risk of these occurring at the onset is vital as any incorrect or inaccurate information that occurs here may well be carried through to all other stages and form the basis for unhelpful interventions.

**Table 2:** Strategies to address the potential presence of confabulation and suggestibility.

Strategy	Illustration	Effect
Utilization of a Structuring Statement	State the purpose of the interview, note that collateral contacts will be utilized, and the interview will conclude with an offer of feedback. Tell them it is okay to say if they do not know information.	A firm structuring statement will help take ambiguity out of the relationship. This will lend to higher accuracy and a decreased likelihood the client feels that they need to deceive or fill in information they do not know.
Foreshadow the upcoming series of information-gathering questions.	*Many of the questions you are about to be asked may feel very personal and too much, but the reason we do this is to collect correct information about you and your life now as well as in the past. We know with this information we can help pick the right things to help you. We ask these same questions of everyone.*	The interview will feel less like an interrogation and more like a conversation. This will contribute to more truthful replies.
Explain how the interview will conclude.	*When we are done talking, I may have to go back and ask a few more questions about things that I forgot to ask earlier.*	
	If there was information gathered that may have been the result of suggestibility, double checking these inconsistencies will mitigate the effect.	
Ask open-ended questions.	*Tell me about the first time you drank some alcohol or smoked some pot. Tell me about what happened after that. Tell me about any drugs and alcohol you have used in your life, before and now.*	Closed-ended questions invite the respondent to give yes/no replies, stifling the conversation. Open-ended questions evoke more from the interviewee.
Ask only one question at a time.	*Tell me about how you get on with your parents.*	Clustering of questions may confuse the interviewee and lead to confabulation of replies. The interviewee may also become overwhelmed, further increasing their difficulty in organizing information.
Use simple, concrete language with short questions.	*Tell me about the jobs you have had including any jobs you have now.*	See above.
Avoid judgment or accusatory phrases.	*What did your parents say about that decision?*	Endeavor to reduce defensiveness throughout the interview.
Remind them that you will be collecting information from other sources to confirm the information they provide.	*To be transparent, know that I will make an effort to confirm as much of the information you provided me through other contacts such as friends, family, employers,* etc.	This will safeguard against inaccurate client replies resulting from suggestibility and/or confabulation.

Beyond a well-crafted structuring statement, the practitioner conducting the risk-need-responsivity assessment should approach the interview as a conversation rather than an interrogation. As Brown and colleagues note, steps can be easily taken when working with individuals with FASD to reduce the risk of inaccuracies associated with traits such as confabulation and suggestibility. Many of these approaches are simple (e.g., the use of open-ended questions, asking only one question at a time, using simple concrete language) but challenging to implement consistently. Finally, to further increase the accuracy of the client responses, even more, due diligence should be given to confirming the responses provided and gathering collateral information. This includes repeating responses back to the interviewee to make sure the response recorded is accurate. Additionally, the assessor should have the interviewee repeat any information provided in their own words to ensure comprehension.

## Summary

Fetal alcohol spectrum disorders are a significantly misunderstood but prevalent problem in the criminal justice system. While 1–10% of the general population meet criteria for an FASD, 17–36% of individuals in the criminal justice system meet the criteria. Many have not yet been identified due to the lack of knowledge about FASD by criminal justice professionals and the lack of adequate screening and assessment. This article provided information to propose a renewed focus on applying and adapting the Risk-Need-Responsivity (RNR) approach to individuals with FASD in criminal justice settings by adequately assessing their needs and adapting interventions following the RNR model for individualized intervention to best meet their needs and reduce recidivism. Despite basic research about the prevalence of FASD in criminal justice populations, further research is needed to explore the reasons for this high prevalence rate, as well as to identify risk/needs assessment, treatment approaches, and interviewing guidelines and their applicability for this highly vulnerable clinical population when encountered in forensic settings.

The Risk-Need-Responsivity Model is a well-established evidence-based approach to lower recidivism with justice involved individuals. The efficacy of the model rests on the three interwoven principles of Risk, Need, and Responsivity. The key to success though lies in the fidelity to each of these principles while accounting for individual differences due to FASD. All three of these principles lose potency if FASD considerations are not taken into consideration in their application. Accurate risk assessments should include FASD-specific symptoms and functioning to ensure the person is not over- or under-supervised which would result in either increased recidivism or the unnecessary use of valuable resources. Both of these supervision outcomes are in contrast to the Risk principle. If inaccurate assessment results in a misattribution of the client’s area of risk, the Need Principle foretells of missed opportunities at treatment and higher recidivism rates. Finally, adherence to the Responsivity principle is maintained by crafting and creating a learning environment which takes into account all the aspects of FASD (see Table 3).

**Table 3:** Fetal alcohol spectrum disorder (FASD) features.

Brain domain of impairment in FASD and central eight impact	Example of possible impact on assessment of risk/need	Example of possible impact on implementation of services (Responsivity)
Executive functioning (may contribute to: criminal history, pro-criminal attitudes, pro-criminal associates, antisocial personality patterns, family relationships, school/work performance, substance abuse, and leisure/ recreation pursuits)	Difficulties in organizing history and thoughts in a coherent and sequential manner, unable to understand the implications of providing certain information, impulsivity may impact on what information is shared and how, agreeing to incorrect information just to get the process over	Difficulties in organizing regular and consistent attendance, impulsivity may impact in a group setting by saying things they later regret or doing things without thinking them through; difficulty planning time and resources to complete homework or other tasks
Adaptive functioning including social skills (may contribute to: criminal history, pro-criminal associates, antisocial personality pattern, family relationships, school/ work pursuits, substance abuse, and leisure/ recreation pursuits)	Difficulties completing paperwork accurately including questionnaires, social desirability effects with the interviewer leading to provision of an inaccurate history, may give an inaccurate account of their skills, abilities, and supports	Difficulties with reading, writing and completing forms, difficulties interacting with other participants in a group setting, highly vulnerable to manipulation and exploitation by others, strong desire to fit in and be accepted by others, focus on being liked and fitting in with peer rather than course content, trying to look knowledgeable/ informed
Cognition (may contribute to: criminal history, pro-criminal attitudes, antisocial personality patterns, school/ work pursuits)	Inaccurate historian of events, difficulties understanding what information is required and relevant Limited understanding of assessment tasks and questions, unable to answer questions in a short-time frame due to slow processing speed, agreeing to things that they do not understand	Lacking problem-solving skills, slow to process and understand information while not identifying this to others, difficulties linking the intervention with other situations outside the intervention (being very concrete), learning not generalizing to other situations; difficulty understanding material/ skills presented Difficulties understanding the requirements of orders, difficulties understanding training or group materials, interventions moving too fast to process information, difficulties with problem-solving novel and unexpected situations
Attention (may contribute to: pro-criminal attitudes, antisocial personality pattern, family relationships, school/ work pursuits, leisure/ recreation pursuits)	Difficulties attending to interview processes leading to leaving out important information, missing key pieces of information about processes leading to reduced comprehension and understanding	Difficulties keeping up with the pace of programs, easily distracted and distractible to others, missing key messages and information, difficulties tracking time for appropriate and consistent attendance
Affect and Emotional Regulation (may contribute to: pro-criminal attitudes, pro-criminal associates, antisocial personality patterns, family relationships, school/ work, substance abuse, leisure/ recreation pursuits)	Difficulty comprehending information and processes due to feeling emotionally overwhelmed, anxiety may contribute to accepting incorrect information or being unable to speak up, anger outbursts when having difficulty understanding or communicating	Difficulty working in groups, difficulties managing emotions when triggered in group setting, mood issues exacerbated by demands
Memory (may contribute to: criminal history, pro-criminal associates, antisocial personality patterns, family relationships, school/ work, leisure/ recreation pursuits)	Inaccurate history giving, tendency to fill in gaps in memory with inaccurate information (i.e., confabulation), heightened suggestibility	Difficulties retaining information and learning materials, vulnerability to suggestions from others, confusion, and frustration
Language (may contribute to: criminal history, pro-criminal attitudes, pro-criminal associates, antisocial personality pattern, family relationships, school/ work, leisure/ recreation pursuits)	Difficulties understanding what is being asked of them or the repercussions of this, difficulty expressing themselves and being able to give comprehensive and accurate answers, saying they understand when they do not, agreeing to things that they do not understand	Difficulties understanding the content of courses or training, failure to speak up about not understanding, excessive talkativeness without substantial content, ability to parrot back information without a deeper understanding or appropriate application
Academic performance (may contribute to: criminal history, pro-criminal associates, school/ work, leisure/ recreation pursuits)	Difficulties reading and writing and completing paperwork associated with assessment	Difficulties with reading and writing in completing courses or training materials, difficulties completing other paperwork associated with the conditions of their orders, becoming frustrated and easily overwhelmed with learning tasks, dropping out or becoming disruptive when tasks become too difficult
Motor Skills (may contribute to: criminal history, school/ work, leisure/ recreation pursuits)	Difficulties with physically completing forms and questionnaires due to poor fine and visuo-motor skills	Difficulties with writing down training materials, non-compliance with completing forms and paperwork, anger and frustration
Brain Structure Abnormalities (may contribute to: school/ work, leisure/ recreation pursuits)	Hearing loss, seizures can interfere with accurate assessment, and visual difficulties	Hearing loss interferes with communication, seizures can interfere with participation in training and interventions, not wanting to speak up and draw attention to themselves, and increased medical needs interfere with attendance
Sensory (may contribute to: criminal history, pro-criminal attitudes, antisocial personality pattern, family relationships, school/ work, substance abuse, leisure/ recreation pursuits)	Giving inaccurate responses due to sensory overload, perceived non-compliance, sensory overstimulation can contribute to emotional dysregulation or difficulty with attention/ concentration	Sensory overload in intervention settings – too much noise, too many people, etc., perceived non-compliance related to distraction, frustration, and withdrawal or aggressive behaviors

The danger of not following RNR principles and accounting for FASD features here is twofold. Even practitioners and agencies which adhere to the principles of RNR with the utmost fidelity fail to adequately capture the risk and needs of clients with FASD if they are not adequately knowledgeable and informed. The current state of training simply does not address this population, nor the complexity their disorder brings to the equation. The inability to accurately assess the FASD clientele then leads to a myriad of case plan shortcomings. Not only does the inaccurate determination of risk level lead to improper supervision of the client, but the change targets being incorporated into the case plan may not have been authoritative.

In summary, the aim of this article has been to review the Risk-Need-Responsivity Model and propose its applicability to offenders diagnosed with FASD. Practical examples of how FASD may impact on each element have been explored as potential areas of challenge and areas for further research have been highlighted. The following represents a summary of the six key points of the article:

1. The Risk-Need-Responsivity (RNR) Model is an evidence-based framework designed to reduce future recidivism in general offender populations.
2. Although applied to those with FASD in practice due to their over-representation in criminal justice settings, little research has been conducted specifically on this population and how the RNR principles may best be applied to those with FASD.
3. Further research is needed to establish a risk assessment process which can accurately and reliably predict recidivism among individuals diagnosed with FASD and specifically take into account the vulnerabilities that arise as a result of their severe neurodevelopmental impairments.
4. Best practice interviewing guidelines for individuals diagnosed with FASD need to be established so that the benefits of increasingly accurate information, including the gathering of collateral information, can be allowed to flow through to the provision of more appropriate and successful interventions.
5. Criminal justice and forensic mental health professionals need to be knowledgeable of and responsive to the cognitive and adaptive impairments associated with prenatal alcohol exposure.
6. Training is critical to the faithful implementation of risk/need assessment and treatment approaches in the general criminal justice population and even more so with the FASD population given their highly complex presentations and heightened vulnerabilities.

## Author contributions

All authors listed have made a substantial, direct, and intellectual contribution to the work and approved it for publication.

## Conflict of interest

The authors declare that the research was conducted in the absence of any commercial or financial relationships that could be construed as a potential conflict of interest.

## Publisher’s note

All claims expressed in this article are solely those of the authors and do not necessarily represent those of their affiliated organizations, or those of the publisher, the editors and the reviewers. Any product that may be evaluated in this article, or claim that may be made by its manufacturer, is not guaranteed or endorsed by the publisher.
